# Nutrition and Athlete Immune Health: New Perspectives on an Old Paradigm

**DOI:** 10.1007/s40279-019-01160-3

**Published:** 2019-11-06

**Authors:** Neil P. Walsh

**Affiliations:** College of Human Sciences, Bangor University, Bangor, LL57 2PZ UK

## Abstract

Respiratory and gastrointestinal infections limit an athlete’s availability to train and compete. To better understand how sick an athlete will become when they have an infection, a paradigm recently adopted from ecological immunology is presented that includes the concepts of immune resistance (the ability to destroy microbes) and immune tolerance (the ability to dampen defence yet control infection at a non-damaging level). This affords a new theoretical perspective on how nutrition may influence athlete immune health; paving the way for focused research efforts on tolerogenic nutritional supplements to reduce the infection burden in athletes. Looking through this new lens clarifies why nutritional supplements targeted at improving immune resistance in athletes show limited benefits: evidence supporting the old paradigm of immune suppression in athletes is lacking. Indeed, there is limited evidence that the dietary practices of athletes suppress immunity, e.g. low-energy availability and train- or sleep-low carbohydrate. It goes without saying, irrespective of the dietary preference (omnivorous, vegetarian), that athletes are recommended to follow a balanced diet to avoid a frank deficiency of a nutrient required for proper immune function. The new theoretical perspective provided sharpens the focus on tolerogenic nutritional supplements shown to reduce the infection burden in athletes, e.g. probiotics, vitamin C and vitamin D. Further research should demonstrate the benefits of candidate tolerogenic supplements to reduce infection in athletes; without blunting training adaptations and without side effects.

## Key Points

**Table:** 

A new paradigm for exercise immunology is presented that considers ‘*resistance’* (the strength of the immune weaponry) and ‘*tolerance’* (the ability to endure microbes and dampen defence activity).
A contemporary view is that immune ‘*resistance’* is not suppressed in athletes under heavy training; as such, it is not surprising that nutritional supplements targeted towards improving immune ‘*resistance*’ show limited benefits to reduce the infection burden in athletes—‘if it ain’t broke, don’t fix it!’
This paradigm of ‘*resistance’* and ‘*tolerance’* helps to explain why nutritional supplements with tolerogenic effects (e.g. probiotics, vitamin C and vitamin D) are the new targets—tolerogenic supplements may reduce the infection burden in athletes.

## Introduction

“If we could give every individual the right amount of nourishment and exercise, not too little and not too much, we would have found the safest way to health.”

*Hippocrates c. 460—377 B.C.*

It has long been known that an individual’s nutritional status influences both their susceptibility to infection and their response to infection in terms of clinical outcome [1, 2]. Leyton’s seminal work in British and Russian prisoners during the Second World War made an important connection between malnutrition and tuberculosis morbidity; at a time predating vaccination for tuberculosis [1]. The emaciated prisoners shared the same living and working conditions, performing at least 12 h of hard manual labour each day on a daily diet providing only ~ 1600 kcal. The British prisoners also received a Red Cross food supplement containing 1300 kcal and 45 g protein each day. Despite the same exposure to infection, a comparative radiographic survey showed a tuberculosis prevalence of 1% in the British prisoners and 19% in the Russian prisoners: tuberculosis onset, development and death were more rapid in the malnourished Russian prisoners. Nutrient availability influences immunity because macro- and micro-nutrients are involved in a multitude of immune processes, e.g. macronutrients are involved in immune cell metabolism and protein synthesis and micronutrients in antioxidant defences. That there might be an important interaction between nutritional status and immune health in athletes under heavy training has received much interest and fervour amongst scientists and practitioners since Shephard and Shek’s landmark review on this subject in 1995 [3].

Inadequate nutrition, specifically low energy availability, has been placed firmly in the spotlight recently as a risk factor for infection in elite athletes [4]. Approximately half of all female athletes in two recent studies were classified as having low energy availability and this was associated with a four to eight times higher risk of upper respiratory infection (URI) in the months preceding the summer Olympics [4, 5]. Although these findings are limited to the level of association (not causation), they raise interest in the role that nutrition may play in maintaining athlete immune health, the focus of this review. The aim of this review is not to provide an exhaustive account of the influence of individual macronutrients and micronutrients on athlete immunity; this can be found elsewhere [6]. Rather, the aim is to provide a new theoretical perspective to improve our understanding of how nutrition may influence athlete immune health and set a path for more focused research efforts moving forward. The review covers important controversies, misunderstandings and paradoxes in the field. First up, to set the scene, recent advancements in our understanding of the infection burden in athletes and the prominent infection risk factors are covered. Upon this backdrop, the evidence that energy deficiency decreases immunity and increases infection in athletes is scrutinised. The overly simplistic and longstanding view held by many that nutritional supplements should be targeted towards countering the apparently weakened immune weaponry (termed ‘resistance’) in otherwise healthy elite athletes is also examined.

A new paradigm for exercise immunology, recently adopted in human immunology from ecological immunology [7], is offered that considers the beneficial tolerogenic interactions between pathogens and the immune system (‘tolerance’ refers to the ability to endure microbes). Looking through this new lens provides a much clearer picture with regard to the rather conflicting and often disappointing findings of studies investigating nutritional supplements and athlete immune health. This new theoretical perspective provides a framework for focused research endeavours on targeted tolerogenic nutritional supplements to reduce the burden of infection in elite athletes.

## Infections Pose a Serious Problem for Athletes

An URI, such as a common cold, might only present an unwelcome nuisance for many of us; however, URI and other infections such as those that affect the gastrointestinal system may limit an elite athlete’s availability to train and take part in major competition [8]. After injury, illness (primarily respiratory but also gastrointestinal) was the second most common reason for an elite athlete to seek medical attention either during training or when competing at the summer or winter Olympic Games [8–10]. In a 3-year surveillance study of 322 Olympic athletes, ~ 70% of illnesses recorded by medical staff resulted in ‘time loss’ (complete absence) from training and competition; the remaining illnesses resulted in ‘performance restriction’ (e.g. reduced volume and/or intensity of training) [8]. Needless to say, sickness absence from training is incompatible with success in elite sport, which demands a consistently high training volume. In accordance with this logic, the empirical evidence shows that medal winners at major sporting events, including the Olympics and World Championships, experience fewer URIs and shorter lasting URIs than less successful, national-level athletes [11, 12]. Furthermore, URI incidence correlates negatively with annual training volume in elite athletes, *viz*. ‘the less sick, the more an athlete can train’ [13].

Against their better judgement, athletes often choose to ignore illness symptoms for fear of missing training and competition [14]; likely they feel that achieving success is down to an ability to suffer and ‘push on’ through adversity, when others would stop. Runners who reported ongoing or recent illness symptoms (in the last 8–12 days) before an endurance race were more likely to drop out of the event; albeit, ~ 98% of runners did reach the finish line [14]. It is a widely held belief that heavy exertion may protract the course of an ongoing infection [15, 16]. Worse still, heavy exertion during, or after incomplete recovery from, a viral infection can result in serious medical complications including myositis, rhabdomyolysis and myopericarditis; the latter of which can cause acute arrhythmias leading to sudden death [17, 18]. Case reports in human casualties [17, 18], corroborated by the findings of animal research [19], indicate that a combination of heavy exertion and viral infection (particularly viruses that cause URI) increases the likelihood of life-threatening myopericarditis; likely either as a consequence of direct viral invasion of the heart or host-mediated inflammatory pathology [18]. A corollary of the host-mediated inflammation and immune perturbations during infection may be altered thermoregulation and an increased risk of life-threatening exertional heat stroke in military personnel and athletes [20]. Although limited to the level of association, case studies of exertional heat illness casualties often report recent infection symptoms [21]. Exertional heat illness is certainly not confined to military settings, or to lesser fit athletes, as half of all elite athletes in one recent survey reported having suffered heat illness symptoms (e.g. vomiting and collapse) and 1 in 12 reported a previous medical diagnosis of exertional heat illness [22]. Mindful of these risks, it cannot be overemphasised how important it is for athletes to wait until all symptoms have cleared before returning to exercise after infection, in line with current recommendations [23]. Clearly, respiratory and gastrointestinal infections pose a problem for high-level athletes; limiting availability for training and competition and in some cases leading to serious medical complications.

### Risk Factors for Infection and Lowered Immunity in Athletes

Scholarly research on this topic began in earnest in the 1980s [24–27], thus it is somewhat surprising that only very recently has research begun to scratch the surface regarding the prominent risk factors for infection in elite athletes [4, 11, 12]. Central to the doctrine of early exercise immunology was the concept that heavy exercise temporarily decreases immunity providing an ‘open window’ for URI and other infections [25, 26]. Periods of overreaching and longer term mal-adaptation (coined ‘overtraining’) were also associated with neuroendocrine modulation, decreased immunity and increased URI [28, 29]. These findings supported the prevailing notion of the time that accumulated training stress compromised immune health and increased infection risk. As such, for many years ‘exercise immunologists’ broadly accepted, and focused their research efforts towards countering heavy exercise as a prominent risk factor for URI in athletes. Interested readers are directed elsewhere for comprehensive accounts of: the inner workings of the immune system [30]; the neuroendocrine modulation of immunity in response to stress [31]; and the influence of heavy exercise on immunity [32, 33] and respiratory infection [34]. In short, both innate and acquired immunity are often observed to decrease transiently during the recovery period after prolonged heavy exertion; typically of the order 15–70% [24, 35–39]. However, whether these transient changes in immunity with acute heavy exercise and intensified training are sufficient to increase URI susceptibility in accordance with the ‘open window’ theory has been in doubt for some time [13, 15, 39–41]. Ekblom et al.’s findings on URI at the 2000 Stockholm marathon provided the first serious challenge to the ‘open window’ theory by showing no increase in URI symptoms post-race [15]; contrasting earlier reports of increased URI after marathons and ultramarathons [27, 42]. In addition, Ekblom et al.’s observations supported the idea that pre-race URI symptoms may have accounted for reports of increased URI after endurance events [15].

Recent research highlights prominent risk factors for infection in elite athletes and military personnel broadly similar to those in the wider population; including, wintertime (common cold and influenza season) [11, 12]; high levels of psychological stress, anxiety and depression [5]; poor sleep (< 6 h per night) [43] and long-haul travel [12]. By contrast, increases in training load resulted in relatively small increases in URI and gastrointestinal infection incidence in one recent study in elite swimmers [11] and no change in infection incidence in another recent study in elite cross-country skiers [12]. Psychological stress, sleep disturbances and physical exertion all influence immunity via activation of the hypothalamic–pituitary–adrenal axis and the sympathetic nervous-system. Common pathways and effector limbs for the body’s response to stress in its various forms give rise to increases in circulating catecholamines and glucocorticoid hormones widely acknowledged to modulate immune function [31]. That poor mental health [5] and poor sleep [43] predict URI in elite athletes and military personnel is in keeping with seminal work, in the wider population, showing dose-response relationships between both psychological stress and the common cold [44] and sleep quantity and quality and the common cold [45] after intra-nasal inoculation with rhinovirus. Likewise, the increased incidence of URI and gastrointestinal infections after long-haul flights in elite athletes [12, 46] is a widely reported phenomenon in occupational travellers [47]. It is quite conceivable that aspects of psychological well-being (e.g. perceived stress and mood) account, at least in part, for the observed alterations in immunity and infection in studies of poor sleep and long-haul travel [33]; and in studies investigating the influence of overreaching and mal-adaptation on immune health in athletes; where depressed mood is a common feature [28, 48]. Indeed, evidence now points to a modulating effect of anxiety and perceived stress on the immune response to exercise [49] and the risk of URI in highly active individuals [50].

## How Does Nutrition Influence Immunity and Infection?

The immune system’s ability to clear viruses, bacteria and other pathogens, termed ‘resistance’, is dependent upon an adequate supply of energy from important fuel sources; including, glucose, amino acids and fatty acids. In addition to fuel requirements, cell proliferation requires nucleotides for DNA and RNA synthesis and amino acids for protein synthesis. An adequate supply of amino acids is also required for the production of proteins such as immunoglobulins, cytokines and acute-phase proteins [51]. The influence of severe restriction of all nutrients (Marasmus) and protein-energy-malnutrition (Kwashiorkor) on immunity and infection-related mortality in developing countries is well documented [52, 53] (see also Sect. 4). Severe energy restriction may also influence immunity via activation of the hypothalamic–pituitary–adrenal axis and increases in stress hormones: cortisol, for example, is widely acknowledged to have anti-inflammatory effects [31]. Micronutrients play important roles in nucleotide and nucleic acid synthesis (e.g. iron, zinc and magnesium) and antioxidant defences that limit tissue damage (e.g. vitamins C and E). Antioxidant availability (e.g. vitamin C) may be particularly important during heavy exertion or infection when oxidative stress increases [54]. Some micronutrients can directly influence immune cell functions by regulating gene expression (e.g. vitamin D) [51, 55].

There are other ways in which nutrition may affect immunity and infection; for example, prebiotics and probiotics may influence immunity indirectly by modifying the gut microbiota [56] and elemental zinc in oral lozenges may directly inhibit viral activity in the oropharyngeal region, with purported therapeutic benefits for URI [57]. Calder highlights the bi-directional link between nutrition, immunity and infection [51]. On the one hand, malnutrition has a well-described negative influence on immunity and resistance to infection; but on the other hand, the widely reported increase in energy requirement during infection paradoxically coincides with reduced appetite (anorexia) and nutrient malabsorption, hitherto a poorly described phenomenon (see Sect. 4).

## Does Energy Deficiency Decrease Immunity and Increase Infection in Athletes?

There has been much debate of late about the influence of energy deficiency on athlete health [58, 59]. Interest in this topic has been stoked by the recent observation that low energy availability was associated with increased illness symptoms in elite female athletes [4, 5]. Besides the obvious limitation that this observation was restricted to female athletes, the authors recognised the need for studies to directly assess energy availability (they used the LEAF questionnaire) and perform measures of immunity and pathology, the latter to confirm infection. These findings are undoubtedly interesting but somewhat at odds with the instructive literature on the influence of anorexia nervosa and protein-energy-malnutrition on immunity and infection; and the findings of studies examining immunity during moderate and severe energy restriction in athletic and non-athletic populations.

Paradoxically, anorexia nervosa is considered to be protective against infection, at least until the condition is extremely severe (body mass index [BMI] < 15 kg/m^2^) [60–62]; moreover, infections are reported to occur readily upon refeeding [63, 64]. Recent research sheds some light on this paradox by showing that anorexia improves immune tolerance and survival during bacterial infection (‘starve a fever …’) yet potentiates the progression and lethality of viral infection (‘… feed a cold’) [65]. Immunity is surprisingly well preserved in patients with anorexia nervosa. Patients tend to have increased infections only in the most advanced states of hospitalisation; typically when ≥ 40% body weight has been lost and there is evidence of decreased cell-mediated immunity (delayed-type hypersensitivity), and decreased humoral immunity (serum immunoglobulins); although the latter is less markedly affected [61, 66]. Analogous are the findings of decreased cellular immunity (lymphoid atrophy, lower T-lymphocyte counts and function, delayed-type hypersensitivity) and increased infections in malnourished children with severely advanced Kwashiorkor (< 70% body weight to height recommendation) [52, 67, 68]. Failure to meet the significantly raised energy demands during infection likely accounts for the poor infectious outcomes in the most severe cases of anorexia nervosa and protein-energy-malnutrition [68]. The key feature of Kwashiorkor, widely recognised to suppress immunity and increase infection incidence, is low protein intake [52]. The well-preserved immunity and robust infection resistance typical in patients with anorexia nervosa is likely because protein intake is relatively sufficient (carbohydrate and fat intake are typically reduced) [69], unlike the situation in starvation where protein deficiency is considered largely responsible for immune suppression, e.g. reduced lymphocyte proliferation [70]. The important influence of dietary protein on immunity has been demonstrated in mice challenged with influenza [71]. Mice fed a very low protein diet exhibited lower virus-specific antibody responses, lower influenza specific CD8^+^ T lymphocyte counts and rapid mortality after influenza infection, compared with an isocaloric adequate protein diet. Importantly, increasing protein intake in the mice fed the very low protein diet improved protective immunity. Turning our attention back to elite female athletes with low energy availability, protein intake appears to be more than adequate to support immunity; typically exceeding both government recommendations (0.8–0.9 g/kg/day) and those proposed for endurance athletes (1.2–1.7 g/kg/day) [72, 73]. For example, in a group of elite female distance runners with low energy availability (BMI 18.9 kg/m^2^), protein intake was 2.1 g/kg/day, and in those with amenorrhea (BMI 18.5 kg/m^2^; absence of menses ≥ 3 months), protein intake was 2.4 g/kg/day [73]. As such, there must be some other explanation for the increased URI reports in elite female athletes with low energy availability. It is conceivable that poor mental health (e.g. stress, anxiety and depression), highly prevalent in female athletes with low energy availability [4, 74, 75], plays a role in the increased URI reports: psychological stress, anxiety and depression have a well-known and marked influence on immunity and infection resistance [44, 76]. Research is required to solve the puzzle that anxiety disorders and depression are also highly comorbid with anorexia nervosa [77, 78], yet this condition appears to provide some protection against URI [60–62].

The findings from randomised controlled trials are also informative in answering the question; does energy deficiency decrease immunity and increase infection in athletes? A 25% calorie restriction, over a 2-year period in non-obese adults (BMI 25.1 kg/m^2^), resulted in a 10% body weight loss but did not decrease in-vivo cell-mediated immunity (skin delayed-type hypersensitivity test) or antibody responses to T- and B-cell-mediated vaccinations (hepatitis A, tetanus/diphtheria and pneumococcal); and had no effect on clinical infections [79]. On the contrary, the authors observed salubrious benefits of moderate calorie restriction, without malnutrition, on inflammation; as demonstrated by 40–50% lower circulating C-reactive protein and tumour necrosis factor-α. These inflammatory molecules have well-established roles in the pathogenesis of multiple chronic diseases and aging; as such, the findings align with the contemporary view that dietary restriction without malnutrition elicits a healthy phenotype and extends the lifespan [79–81]. Of course, the findings of research examining the influence of long-term moderate energy deficits on immunity in non-obese adults are interesting but arguably of limited relevance to athletic populations. For example, there are obvious population differences in body composition (average BMI of 25.1 kg/m^2^ is considered overweight [79]) and the energy deficit in athletic populations typically results from high energy expenditure during heavy training, rather than calorie restriction. Notwithstanding, studies in athletic and military populations investigating short-term severe energy restriction (48 h, ~ 90% restriction) and long-term moderate energy restriction during training (8 weeks, ~ 25% restriction) show only subtle and short-lived changes in immunity [82–84]; and no increase in the immune-modulating hormone, cortisol. Concordant with these findings, low energy availability in female and male endurance athletes has little effect on circulating cortisol [85]. This is perhaps not surprising as a recent meta-analysis showed that circulating cortisol increases in states of complete fasting but typically not during less severe energy restriction [86]. It is a common misconception, and an oversimplification, that increases in circulating cortisol always decrease immunity [87]; the reality is more nuanced. For example, increases in circulating cortisol during short-term stress (lasting minutes to hours) can have adjuvant-like effects that enhance immunity [31, 88]. In contrast, chronic stress (lasting days to months) can disrupt the cortisol circadian rhythm and increase glucocorticoid resistance, with harmful effects on immunity, inflammation and infection resistance [31, 89]. In summary, direct evidence to support the notion that energy deficiency of the magnitude often reported in elite female athletes compromises immunity is currently lacking.

## New Theoretical Perspective on Nutrition and Athlete Immune Health

**Fig. 1:**
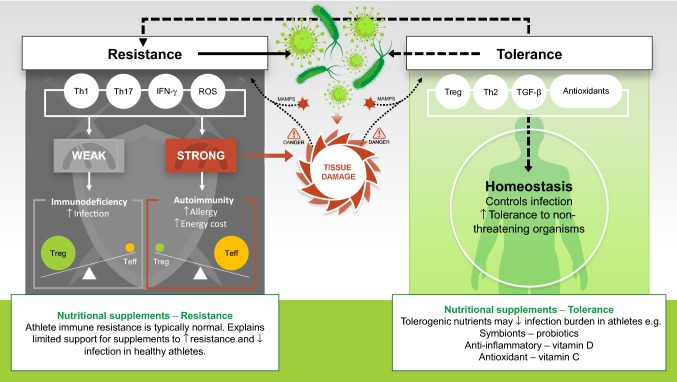
Model of resistance and tolerance in host-pathogen interactions: value of nutritional supplementation. Dark shaded area on the left (arrows with solid lines) shows classical view of immune ‘resistance’ where the immune weaponry protects the host by attempting to reduce the pathogen burden, e.g. through cell-mediated killing and release of reactive oxygen species (ROS). Weak resistance results in immunodeficiency and increased risk of infection. In contrast, an overly exuberant immune response to a pathogen causes tissue damage and wasteful diversion of energy resources away from other important functions. An overly strong immune response is associated with autoimmunity and allergy. In this simple model, homeostasis is achieved by balancing effector and regulatory sides of the scales. This classical model of immune homeostasis overlooks important tolerogenic interactions with the pathogen. The concept of ‘tolerance’, the ability to endure microbes, (light green shaded area on the right and arrows with broken lines) has been adopted from ecological immunology where work in invertebrates shows important tolerogenic interactions between the host and microbes, the findings of which are generalisable to vertebrates [91, 93]. Pathogens influence the magnitude of the immune response by displaying microbe-associated molecular patterns (MAMPS) and by stimulating the release of danger signals from damaged tissue. Tolerance in this model dampens defence activity (upper broken arrow) yet controls infection at a non-damaging level, with the added benefit of a lower energy cost. This explains how we tolerate commensal bacteria rather than eliciting an immune response to obliterate the large abundance of bacteria in the gut. This model also helps to explain why nutritional supplements with tolerogenic effects may reduce the burden of infection (e.g. reduced severity and duration) in otherwise healthy athletes. *IFN-γ* interferon gamma, *Teff* effector T cells, *TGF-β* transforming growth factor-beta, *Th* T-helper lymphocyte, *Treg* regulatory T cells

Traditionally, immunologists have focused their efforts on understanding the immune weaponry at our disposal in the fight against infectious pathogens (termed ‘resistance’). Ecological immunologists prefer a model describing not only resistance but also ‘tolerance’, defined as the ability to endure a microbe [7, 90]. Ayres and Schneider elegantly describe a paradigm using these concepts ‘resistance’ and ‘tolerance’ to better understand human-pathogen interactions [91]. Using a castle metaphor, they describe the inhabitants of the fortress performing various tasks; including, repairing the walls, raising offspring and distributing food. At the same time, the inhabitants must decide whether a battle is worth fighting and the appropriate weapons to use: the immune equivalent of ‘choosing your battles wisely’. Key to effective tolerance is a proportionate immune response: an overly exuberant immune response can cause excessive tissue damage and unnecessarily allocate energy resources away from vital functions; vice-versa, a weak immune response increases susceptibility to damage from the pathogen (Fig. 1) [91].

Reactive oxygen species play an important role in host defence against infection but increased oxidative stress during an immune reponse can result in collateral tissue damage, placing an increased demand on antioxidant scavenging during infection. Seminal research in bumblebees has demonstrated the cost of full-blown immune activation for host survival: starvation significantly decreased survival time in immune-activated compared with immune-naïve bumblebees [92]. Given the tissue damage and increased energy cost during a full-blown immune response, the immune system has likely evolved to control persistent infection at a non-damaging level and exhibit tolerance to non-threatening organisms (Fig. 1) [7, 91, 93]. A prime example is the mutualistic bacteria that reside in the gut; the immune system does not raise a pathogenic response to obliterate the grams of lipopolysaccharide in the intestinal lumen [94]. Co-stimulation, by microbe-associated molecular patterns (MAMPS) and danger signals from damaged tissue, is required for full immune activation. In addition, the localisation of MAMPS and associated pattern recognition receptors at the base of intestinal crypts limits contact with non-pathogenic microbes; together, co-stimulation and spatial localisation of MAMPS facilitate host tolerance to mutualistic bacteria that reside in the gut [91, 93, 95]. Evolution has preferentially selected non-toxic effectors and receptors, in accordance with this model of tolerance; for example, antimicrobial peptides are more toxic to pathogens than to self and Toll-like receptors have a higher affinity for pathogen-associated molecules [94]. Homeostasis is achieved by an appropriate balance of resistance and tolerance that allows us to fight infection, where the signals indicate this is necessary, yet maintain a healthy relationship with the mutualistic bacteria in our gut (Fig. 1).

This new theoretical perspective may improve our understanding of how sick we will become when we have an infection (in terms of severity and duration) [7], and more clearly elucidate a role for nutrition, particularly in terms of tolerance (Fig. 1, Sect. 5.2). Of course, it stands to reason that a frank deficiency of a nutrient required for proper immune function will decrease immune resistance and increase susceptibility to infection. Examples include the well-known influence of dietary protein deficiency on host defence (Sect. 4) [52, 70, 71] and evidence that a frank deficiency in zinc decreases immunity [96]. However, growing evidence indicates that for some nutrients there are times when intakes above recommended levels may have beneficial effects on immunity [97]; likely by optimising the delicate balance between resistance and tolerance.

Looking through this new lens, illustrated in Fig. 1, brings into sharp focus the hitherto rather mixed picture presented by studies investigating nutritional supplements and athlete immune health. For example, this model helps to explain why nutritional supplements with tolerogenic effects may reduce the burden of infection in otherwise healthy athletes (e.g. reduced severity and duration) (Sect. 5.2). Clearly, its no longer sufficient to ask only if a nutritional intervention will stop the athlete getting sick; perhaps its more pertinent to ask, will the nutritional intervention reduce how sick the athlete will get?

### Nutritional Supplements for Immune Resistance: If it Ain’t Broke, Don’t Fix it!

As logic would dictate, support for nutritional supplements to improve immune resistance (and thus decrease pathogen burden) comes largely from studies in those with impaired immunity, such as the frail elderly and clinical patients; particularly in those with poor nutritional status [51, 97]. Over the last 25 years or so, exercise immunologists have actively researched nutritional supplements to improve immune resistance in athletes (Table 1). For much of this period, there was a broad acceptance amongst exercise immunologists that immunity was impaired in athletes under heavy training; prompting the search for nutritional countermeasures [98, 99]. A more contemporary view is that the evidence supporting immunosuppression in athletes is lacking [39, 41]. Thus, it is not surprising that supplements targeted towards immune resistance show limited benefits for athlete immunity and host defence: the phrase ‘if it ain’t broke, don’t fix it’ comes to mind (Table 1). One exception is the therapeutic effect of zinc lozenges for treating the common cold. A recent meta-analysis showed that zinc lozenges (75 mg/day of elemental zinc) reduced URI duration by ~ 3 days (33%) when taken < 24 h after the onset of symptoms, and for the duration of the illness [57]. Hemilä points out that the optimal zinc lozenge dosage and composition need to be determined; indeed, many over-the-counter lozenges contain too little zinc or contain substances that bind zinc [57].

Although the exact mechanism(s) require elucidation, zinc may act as an antiviral agent by increasing interferon gamma and decreasing the docking of common cold viruses with binding sites; the latter by decreasing levels of intercellular adhesion molecule-1 [100, 101]. The therapeutic effects of zinc lozenges for treating URI have also been ascribed to antioxidant and anti-inflammatory properties of elemental zinc in the lozenge [102]; as such, zinc lozenges may also have tolerogenic effects on immunity.

**Table 1:** Nutritional supplements and immune resistance^a^ in athletes: proposed mechanism of action and evidence for efficacy

Supplement^b^	Proposed mechanism of action	Evidence for efficacy^c^	References
Zinc	Zinc is required for DNA synthesis and as an enzyme cofactor for immune cells. RNI is 7 mg/day for women and 9.5 mg/day for men. Zinc deficiency results in impaired immunity (e.g. lymphoid atrophy) and zinc deficiency is not uncommon in athletes	*No support* for ‘preventing URI’. Regular high-dose zinc supplementation can decrease immune function and should be avoided	[96, 103]
	Antiviral effects of zinc lozenges	*Strong support* for ‘treating URI’. Meta-analysis shows benefit of zinc lozenges (75 mg/day of elemental zinc) to shorten common cold by ~ 33%; zinc must be taken < 24 h after onset of URI. Many over-the-counter lozenges have too low zinc or contain substances that bind zinc. Optimal lozenge composition and dosage to be determined. Side effects include bad taste and nausea	[57, 104]
Glutamine	Non-essential amino acid that is an important energy substrate for immune cells, particularly lymphocytes. Circulating glutamine is lowered after prolonged exercise and very heavy training	*Limited support* Some evidence of a reduction in URI incidence after endurance events in competitors receiving glutamine supplementation (2 × 5 g). Mechanism for therapeutic effect requires investigation. Supplementation before and after exercise does not alter immune function	[6, 105, 106]
Carbohydrate (drinks, gels)	Maintains blood glucose during exercise, lowers stress hormones and thus counters immune dysfunction	*Limited support* Ingestion of carbohydrate (30–60 g/h) attenuates stress hormone and some, but not all, immune perturbations during exercise. Very limited evidence that this modifies infection risk in athletes	[107–110]
Bovine colostrum	First milk of the cow that contains antibodies, growth factors and cytokines. Claimed to improve mucosal immunity and increase resistance to infection	*Limited support* shows that bovine colostrum blunts the decrease in mucosal immunity and in-vivo immunity after heavy exercise. Some evidence in small numbers of participants that bovine colostrum decreases URI incidence. Further support required	[111–114]
β-Glucans	Polysaccharides derived from the cell walls of yeast, fungi, algae and oats that stimulate innate immunity	*Limited support* Effective in mice inoculated with influenza virus; however, studies with athletes show no benefit to immunity and equivocal findings for risk of URI	[115–117]
Echinacea	Herbal extract claimed to enhance immunity via stimulatory effects on macrophages. There is some in-vitro evidence for this	*Limited support* Meta-analysis shows a small reduction in URI incidence but no influence on URI duration in the general population. Ambiguous findings from a small number of studies in athletes. Further support required	[6, 118]
Caffeine	Stimulant found in a variety of foods and drinks (e.g. coffee and sports drinks). Caffeine is an adenosine receptor antagonist and immune cells express adenosine receptors	*Limited support* Evidence that caffeine supplementation activates lymphocytes and attenuates the fall in neutrophil function after exercise. Efficacy for altering risk of URI in athletes remains unknown	[119, 120]

*RNI* reference nutrient intake, *URI* upper respiratory infection

^a^Resistance reduces the pathogen burden e.g. immune weaponry protects the host

^b^Supplement must come from a reliable source and be tested by established quality assurance programme [121]

^c^Readers are directed to the consensus statement of The International Society of Exercise Immunology for further discussion regarding the evidence for efficacy of these supplements [6]

### Tolerogenic Nutritional Supplements: The New Targets

Tolerance in this model dampens defence activity yet effectively controls infection at a non-damaging level; it also facilitates homeostatic regulation of beneficial intestinal microbial communities (Fig. 1). Looking through this lens it is easy to see why studies involving nutritional supplements with tolerogenic properties have yielded some positive effects for reducing the burden of infection in otherwise healthy athletes (Table 2). Probiotics (and prebiotics) may have tolerogenic effects by influencing intestinal microbial communities and the common mucosal immune system [56]; the antioxidant effects of vitamin C and the anti-inflammatory effects of vitamin D may improve tolerance, mitigating against excessive tissue damage during infection [54, 55, 91]. As mentioned previously, the therapeutic effects of zinc lozenges for treating the common cold, although principally considered to reduce the pathogen burden (improved resistance), have also been attributed to antioxidant and anti-inflammatory (tolerogenic) properties of zinc [102].

**Table 2:** Nutritional supplements for improving immune tolerance^a^ in athletes: proposed mechanism of action and evidence for efficacy

Supplement^b^	Proposed mechanism of action	Evidence for efficacy^c^	References
Probiotics	Mutualist symbiont. Probiotics are live microorganisms that when administered orally for several weeks, can increase the numbers of beneficial gut bacteria. Associated with a range of potential benefits to gut health and tolerogenic effects. Prebiotics are typically non-digestible carbohydrates that increase beneficial gut bacteria	*Moderate-strong support in athletes* with a daily dose of ~ 10^10^ live bacteria; Cochrane review of 12 studies (*n* = 3720) shows ~ 50% decrease in URI incidence and ~ 2 days shortening of URI; minor side effects. More evidence is required supporting probiotic efficacy to reduce gastrointestinal distress and infection, e.g. in travellers’ diarrhoea. Limited support for prebiotics to decrease risk of URI in athletes	[122–124]
Vitamin C	Antioxidant. An essential water-soluble antioxidant vitamin that quenches ROS. RNI is 40 mg/day (UK)	*Strong support for ‘preventing URI’ in athletes* Cochrane review of 5 studies in heavy exercisers (*n* = 598) shows an ~ 50% decrease in URI incidence when taking vitamin C (0.25–1.0 g/day). No reported side effects. However, unclear if antioxidants blunt adaptation in well-trained athletes	[54, 125]
		High vitamin C doses (gram doses) likely required if initiating vitamin C supplementation after onset of URI to compensate for increased inflammatory response. High vitamin C doses during URI have been shown to reduce URI duration. Further research required	
Vitamin D	Anti-inflammatory. An essential fat-soluble vitamin known to influence several aspects of immunity (e.g. expression of antimicrobial proteins). Skin exposure to sunlight accounts for 90% of the annual source of vitamin D. RNI is 5–15 µg/day	*Moderate-strong support* Evidence for deficiency in some athletes and soldiers, particularly in the winter (decreased skin sunlight exposure). Deficiency has been associated with increased risk of URI. Meta-analysis (*n* = 10,933) shows some benefit of supplementation to decrease URI incidence. Recommend monitoring and 1000 IU/day of D_3_ autumn-spring to maintain sufficiency where necessary. Increased risk of adverse outcomes supplementing > 4000 IU/day of D_3_	[55, 126–128]
Polyphenols, e.g. quercetin	Anti-inflammatory and antioxidant. Plant flavonoids. In-vitro studies show strong anti-inflammatory, antioxidant and anti-pathogenic effects	*Low-moderate support* Some evidence of reduction in URI incidence during short periods of intensified training; albeit, in small numbers of untrained subjects. Limited influence on markers of immunity. Putative anti-viral effect for quercetin. Further support required	[129, 130]
Omega-3 PUFAs	Anti-inflammatory. Found in fish oil. Claimed to exert anti-inflammatory effects post-exercise by regulating eicosanoid formation, e.g. prostaglandin. Prostaglandin is immunosuppressive	*Limited support* for blunting inflammation and functional changes after muscle damaging eccentric exercise in humans and no evidence of reducing risk of URI in athletes. Some evidence oxidative stress actually increased in athletes supplementing n-3 PUFA	[131–133]
Vitamin E	Antioxidant. An essential fat-soluble antioxidant vitamin that quenches exercise-induced ROS	*No support in athletes* Improved in-vivo immunity and reduced URI incidence in the frail elderly but no benefit in young healthy humans. One study actually showed that vitamin E (and β-carotene) supplementation increased the risk of URI in those under heavy exertion. High doses may even be pro-oxidative	[134–136]

*PUFAs* polyunsaturated fatty acids, *RNI* reference nutrient intake, *ROS* reactive oxygen species, *URI* upper respiratory infection

^a^Tolerance dampens defence activity yet controls infection at a non-damaging level

^b^Supplement must come from a reliable source and be tested by established quality assurance programme [121]

^c^Readers are directed to the consensus statement of The International Society of Exercise Immunology for further discussion regarding the evidence for efficacy of these supplements [6]

#### Probiotics

Probiotics are live microorganisms that when administered regularly and in adequate amounts are thought to confer a health benefit on the host by modulating gut-dwelling bacteria (the microbiota) and immunity [137]. There are various mechanisms by which probiotics are purported to benefit immunity and infection resistance, particularly respiratory and gastrointestinal infections; however, thus far these have not been clearly elucidated [97]. Probiotics can improve immune resistance by reinforcing the intestinal barrier and competing with pathogens for both attachment to the gut epithelium and for available nutrients. The products of probiotic metabolism (e.g. lactic acid) can also inhibit pathogen growth in the gut [138]. Probiotics are considered to have important mutualistic benefits for immune health that extend beyond the gut; these interactions between the commensal microbial community and the host immune system occur via the common mucosal immune system [139]. There is now broad agreement that probiotics exert important anti-inflammatory ‘tolerogenic’ effects that maintain homeostasis (Fig. 1); for example, probiotics may prevent unnecessary inflammatory responses to harmless foreign substances in the gut [97].

Results from studies investigating the influence of probiotics on athlete immune health are promising and interested readers are directed to extensive reviews on the subject [56, 139]. One placebo-controlled cross-over trial in 20 elite distance runners showed that probiotic supplementation (*Lactobacillus fermentum*) for 28 days reduced the number of days of URI and the severity of URI symptoms [140]. Another randomised placebo-controlled trial in 64 university athletes reported a lower incidence of URI during a 4-month winter training period in athletes receiving a daily probiotic (*Lactobacillus casei Shirota*) compared with placebo; this study also reported better maintenance of saliva secretory immunoglobulin-A in the probiotic group [122]. Four weeks of supplementation with a multi-species probiotic formulation (*Lactobacillus, Bifidobacterium* and *Streptococcus*) reduced markers of gut permeability and symptoms of gastrointestinal discomfort during exercise-heat-stress [141]. The conclusions of another study were that supplementing marathon runners (*n* = 61) with *Lactobacillus rhamnosus* daily (vs. placebo *n* = 58) for 3 months before a marathon led to shorter lasting gastrointestinal symptoms during the 2 weeks after the race (1 vs. 2.3 days in placebo) [142]. Caution is needed when interpreting these findings as the percentage of runners who actually experienced gastrointestinal symptom episodes during the 2 weeks after the marathon was, as one might expect, low (4% in each group). As such, the sample the authors base their rather speculative conclusion on was small (only two to three runners in each group) [30]. Whether probiotics and prebiotics can prevent travellers’ diarrhoea remains unclear and there is some evidence that prophylaxis is dependent upon the strain of probiotic given [123]. Notwithstanding, results from general population studies show some beneficial effects of probiotics on URI (Table 2). A recent meta-analysis showed that probiotic supplementation reduced the incidence of URI by approximately half, shortened URI duration by approximately 2 days, reduced antibiotic prescription rates and resulted in only minor side effects [124]. However, only 12 studies were included in the meta-analysis (*n* = 3720) and the quality of evidence was rated as low; limitations included the relatively small sample sizes, poor controls and unclear procedures for randomisation. Although the available evidence supporting probiotics to reduce the infection burden in athletes is by no means definitive, studies to date indicate some benefit with little evidence of harm. Athletes might therefore consider probiotic supplementation particularly during periods of increased URI risk, e.g. in the weeks before and during foreign travel [139].

#### Vitamin C

Vitamin C (ascorbic acid) is a major water-soluble antioxidant that is effective as a scavenger of reactive oxygen species in both intracellular and extracellular fluids. Good sources of vitamin C include fruit and vegetables and the reference nutrient intake for adults is 40 mg/day (UK). Vitamin C is found in high concentrations in leucocytes but the level falls dramatically during a common cold, when oxidative stress increases [143]. As such, there is a a sound scientific basis for vitamin C supplementation to improve tolerance by mitigating against excessive tissue damage during infection [91]. There is also a strong rationale for anticipating benefits to reduce URI in athletes who experience increased oxidative stress during heavy exercise [144]. A recent review and a meta-analysis have examined the evidence that daily doses of vitamin C of more than 200 mg have prophylactic and therapeutic effects for the common cold [54, 125]. In a subgroup of five placebo-controlled trials in heavy exercisers (*n* = 598), including marathon runners, skiers and soldiers, vitamin C (0.25–1.0 g/day) decreased URI incidence by 52% [125]. For example, in a double-blind placebo-controlled design, Peters and colleagues showed that 600 mg/day of vitamin C for 3 weeks prior to a 90-km ultramarathon reduced the incidence of URI symptoms in the 2-week post-race period (33% vs. 68% in age- and sex-matched control runners) [145]. Whether the observed benefit of vitamin C for preventing URI symptoms in those under heavy exertion represents a real decrease in respiratory viral infection is an important avenue for inquiry. The rather high URI symptom incidence in the Peters et al. study (68% in placebo) and the observed benefit of vitamin C might relate to exercise-induced bronchoconstriction caused by airway inflammation and injury, which is common during heavy exercise [146, 147]. Regardless of the mechanism, there are clear benefits of vitamin C supplementation (0.25–1.0 g/day) to reduce URI symptoms in athletes (Table 2).

Determining whether initiating vitamin C supplementation after the onset of URI has therapeutic effects is complicated by methodological differences between studies, e.g. differences in the timing of initiating vitamin C supplementation and differences in the duration and dosage of supplementation [125]. Higher daily vitamin C doses may be required for treating URI; for example, 200 mg/day of vitamin C was insufficient to restore leukocyte vitamin C levels during URI but at 6 g/day the decline in vitamin C levels was abolished [143]. Providing 8 g of vitamin C on the first day of a URI shortened URI duration more than 4 g [148] and dose-dependent benefits were shown in another trial providing 3 g/day and 6 g/day of vitamin C [149]. Hemilä et al. suggest that future therapeutic trials in adults should use doses that exceed 8 g/day of vitamin C [125].

One area of uncertainty is whether regular high-dose vitamin C supplementation (1 g/day) blunts some of the adaptations to endurance training [150, 151]. The authors of one study caution against high-dose antioxidant supplementation during endurance training to avoid blunting cellular adaptations [152]. However, whether high-dose antioxidant supplementation blunts training adaptations in highly trained athletes has been questioned [153]. As vitamin C supplementation (0.25–1.0 g/day) is cheap, safe and can prevent URI symptoms in those under heavy exertion, athletes should consider vitamin C supplementation during periods of heightened infection risk, e.g. foreign travel for important competition.

#### Vitamin D

In 1981, the British general practitioner and celebrated epidemiologist, R. Edgar Hope-Simpson was the first to hypothesise that respiratory viral infections (e.g. epidemic influenza) have a ‘seasonal stimulus’ intimately associated with solar radiation. The nature of this ‘seasonal stimulus’ remained undiscovered until the important immunomodulatory effects of the sunlight-dependent secosteroid vitamin D were fully recognised [154, 155]. Vitamin D production as a result of sunlight ultraviolet B radiation penetrating the skin typically provides 80–100% of the body’s vitamin D requirements, with a small amount typically coming from the diet (good sources include oily fish and egg yolks). The recommended daily dietary intake of vitamin D for adults (5 µg or 200 IU in the European Union and 15 µg or 600 IU in the USA) assumes that no synthesis occurs and all of a person’s vitamin D is from food intake, although that will rarely occur in practice [55]. It is now clear that vitamin D has important roles beyond its well-known effects on calcium and bone homeostasis. Immune cells express the vitamin D receptor, including antigen-presenting cells, T cells and B cells, and these cells are all capable of synthesising the biologically active vitamin D metabolite, 1,25-hydroxy vitamin D. It is widely accepted that vitamin D plays an important role in enhancing innate immunity via the induction of antimicrobial proteins; yet many of the actions of vitamin D on acquired immunity are anti-inflammatory in nature. Tolerogenic effects of vitamin D (Fig. 1) prevent overly exuberant immune responses following T-cell activation (e.g. 1,25-hydroxy vitamin D induces development of regulatory T cells and inhibits production of interferon-gamma) [55]. There has been growing interest in the benefits of supplementing vitamin D as studies report vitamin D insufficiency (circulating 25(OH)D < 50 nmol/L) in more than half of all athletes and military personnel tested during the winter, when skin sunlight ultraviolet B is negligible [156, 157]. The overwhelming evidence supports avoiding vitamin D deficiency (circulating 25(OH)D < 30 nmol/L) to maintain immunity and reduce the burden of URI in the general population, athletes and military personnel [158–160]. A recent meta-analysis reported protective effects of oral vitamin D supplementation on respiratory infection (odds ratio 0.88); particularly in those deficient for vitamin D at baseline (odds ratio 0.30) and in those who received oral vitamin D daily or weekly, but not in those receiving one or more large boluses [126]. Vitamin D sufficiency can be achieved by safe sunlight exposure in the summer [127, 161, 162] and where screening indicates insufficiency, 1000 IU/day vitamin D_3_ supplementation in the winter (Table 2) [55, 127].

## Conclusions

This review provides a new theoretical perspective on how nutrition influences athlete immune health. A paradigm recently adopted from ecological immunology is presented that includes immune resistance (ability to destroy microbes) and immune tolerance (ability to dampen an immune response and control infection at a non-damaging level). Through this new lens, it is easy to see why studies investigating nutritional supplements targeted at improving immune resistance in athletes show limited benefits: evidence supporting immune suppression in athletes is lacking; *viz*. if it ain’t broke, don’t fix it! This new perspective sharpens the focus on nutritional supplements with beneficial tolerogenic properties that reduce the infection burden in otherwise healthy athletes; including, probiotics, vitamin C and vitamin D. Further research is required to demonstrate the benefits of candidate tolerogenic nutritional supplements to reduce the infection burden in athletes; without blunting training adaptations and without side effects. When considering nutritional supplementation, athletes must check the supplement comes from a reliable source and is tested by an established quality assurance programme [121].
